# A gastrointestinal stromal tumor found in perforated Meckel’s diverticulum

**DOI:** 10.1186/s40792-016-0196-8

**Published:** 2016-06-29

**Authors:** Shin Miyata, David W. Bliss

**Affiliations:** Children’s Hospital Los Angeles, 4650 W Sunset Blvd, Los Angeles, CA 90027 USA; Arrowhead Regional Medical Center, 400 N Pepper Ave, Colton, CA 92324 USA; 23415S Vermont Ave, Unit C, Torrance, CA 90502 USA

**Keywords:** Meckel’s diverticulum, Gastrointestinal stromal tumor, GIST, Pneumoperitoneum, Perforation

## Abstract

**Background:**

Meckel’s diverticulum is the most common anomaly of the gastrointestinal tract. It is usually asymptomatic, but approximately 4 % present with complications such as bleeding, intestinal obstruction, and inflammation, while perforation is rare. Carcinoid or gastrointestinal stromal tumors are occasionally found in the resected specimens of Meckel’s diverticulum, particularly in the context of perforation.

**Case presentation:**

A 62-year-old male with a recent history of admission and evaluation for hematochezia presented with abdominal pain. His physical examination was consistent with peritonitis. Results of laboratory testing were significant for white blood cell count of 32,000/μL. CT scan of the abdomen revealed pneumoperitoneum. During the exploratory laparotomy, perforated Meckel’s diverticulum was encountered and segmental bowel resection was performed. Histological examination findings were compatible with gastrointestinal stromal tumor within Meckel’s diverticulum.

**Conclusions:**

While gastrointestinal stromal tumor is a rare finding in Meckel’s diverticula, the potential for the coexistence of this and other tumors suggests that segmental resection of the small bowel should be considered in the treatment of perforated Meckel’s diverticulum.

## Background

Meckel’s diverticulum is the most common anomaly of the gastrointestinal tract. It is usually asymptomatic, but approximately 4 % are symptomatic with complications such as bleeding, intestinal obstruction, and inflammation [1]. Diverticulitis, present in 20 % of patients with symptomatic Meckel’s diverticula, may lead to perforation. Most reported cases of perforated Meckel’s diverticulum are secondary to foreign body [2, 3] and spontaneous inflammation [4, 5]. However, cases exist where tumors such as carcinoid or gastrointestinal stromal tumors (GIST) are found in the resected specimen of perforated or symptomatic Meckel’s diverticulum [6–13]. Perforation in combination with another complication is even more rare [14–16]. We report a case of perforated Meckel’s diverticulum in an adult patient with a recent history of hematochezia with unknown source. Pathologic examination of the resected segment of the small bowel revealed a GIST within Meckel’s diverticulum.

## Case presentation

**Fig. 1:**
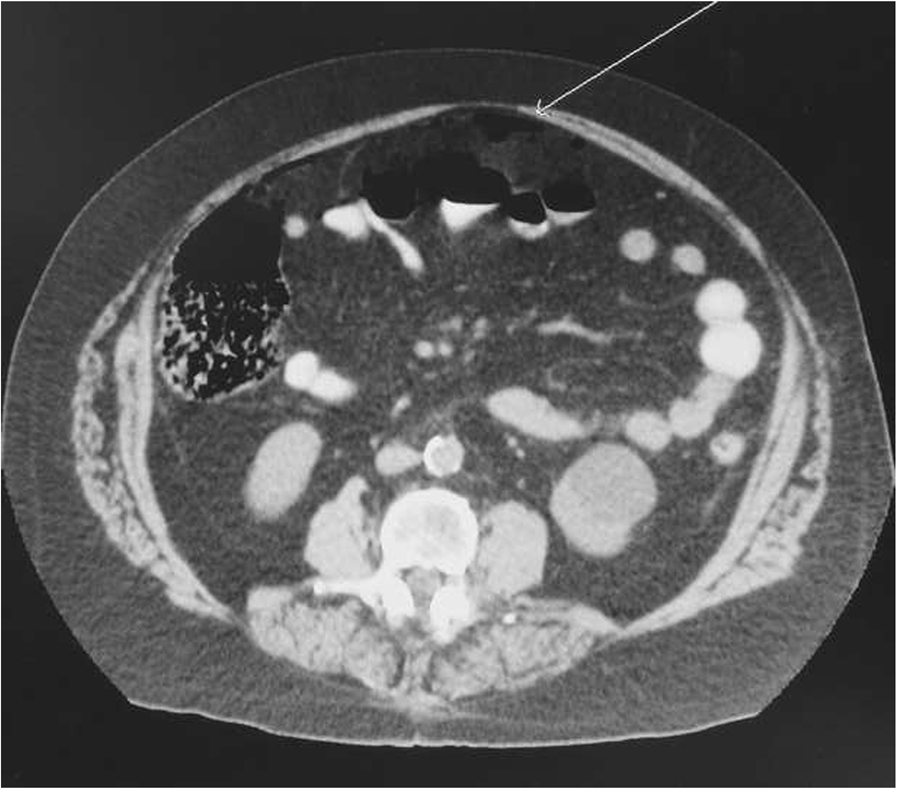
CT scan of the abdomen. Pneumoperitoneum identified on CT scan of the abdomen

A 62-year-old Caucasian man with a recent history of admission and evaluation for hematochezia presented to the emergency department with acute-onset generalized abdominal pain. Prior medical evaluation including upper and lower endoscopy revealed no identifiable source of bleeding. He was then discharged with a capsule endoscopy scheduled as an outpatient but returned when he developed an acute-onset generalized abdominal pain. His physical examination was remarkable for peritonitis with moderate abdominal distention and guarding. His vital signs were normal except for mild tachycardia. Results of laboratory testing were significant for white blood cell count of 32,000/μL without bandemia. CT scan of the abdomen revealed pneumoperitoneum (Fig. 1).

### Operative findings

**Fig. 2:**
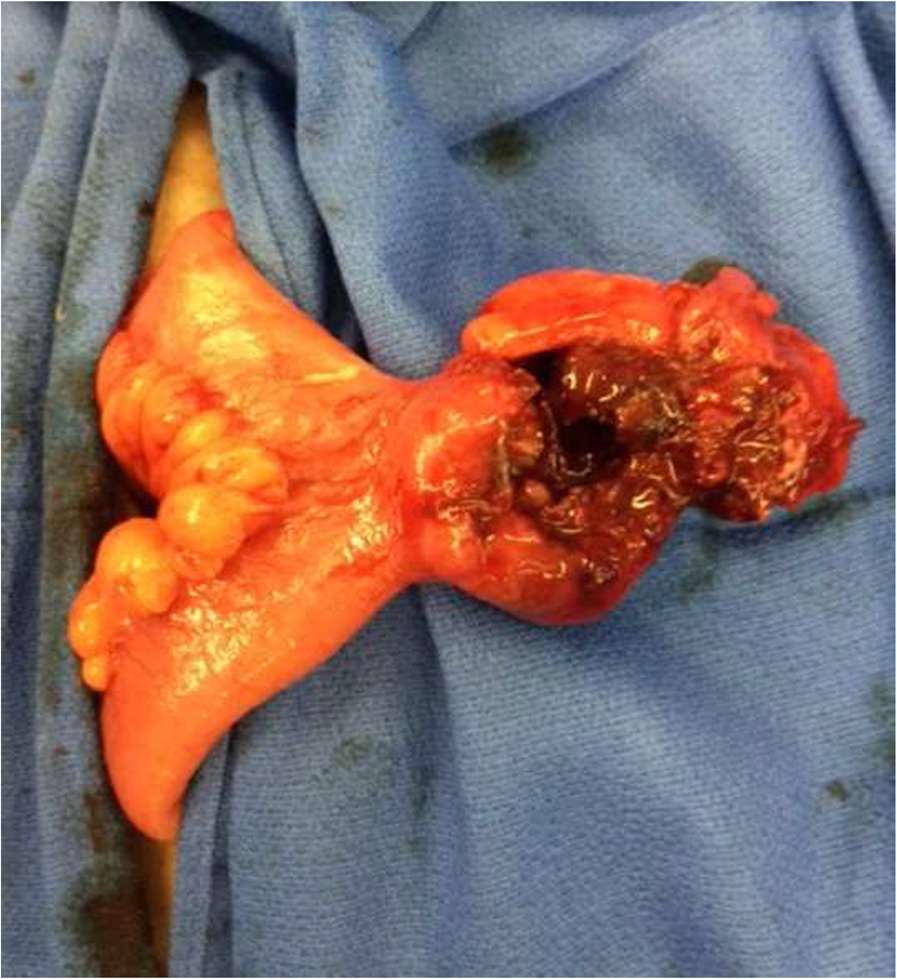
Intraoperative image. Perforated Meckel’s diverticulum

At exploratory laparotomy, we encountered a small amount of murky ascites and a perforated small bowel diverticulum on the anti-mesenteric side of the ileum (Fig. 2), approximately 50 cm from the ileocecal valve. In addition, a fibrous vitelline attachment was found between the diverticulum and the abdominal wall. Segmental small bowel resection with a primary anastomosis was performed. The peritoneal cavity was irrigated with copious amounts of normal saline prior to abdominal closure.

### Pathological findings

**Fig. 3:**
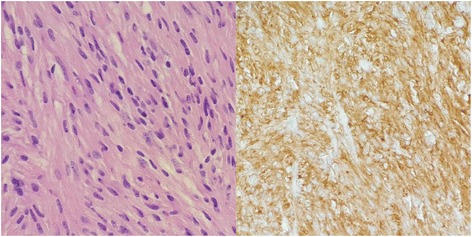
Pathological findings. *Left*—hematoxylin and eosin stain (×40) showing spindle cells with nuclear pleomorphism. *Right*—immunohistochemical stain positive for CD117 (c-kit)

Histological examination revealed a 4-cm, intramural spindle cell neoplasm with moderate nuclear pleomorphism (Fig. 3, left). Immunohistochemical stains for vimentin, SMA, CD34, and CD117 (Fig. 3, right) were positive. The proliferation rate was less than 1 % by Ki-67. No mitotic figures were found in 50 high-power fields. This combination of findings is compatible with GIST.

### Postoperative course

His postoperative course was uneventful. He was discharged home on postoperative day 4. Adjuvant therapy with imatinib was initiated for prevention of tumor recurrence.

## Discussion

Perforation is an unusual clinical manifestation of Meckel’s diverticulum. Most reported cases of perforated Meckel’s diverticulum are secondary to foreign body [2, 3] or spontaneous perforation due to diverticulitis [4, 5]. Tumors found in Meckel’s diverticulum are also rare (0.5–3.2 %) [6], with carcinoid being the most common. To date, only 15 cases of GIST arising in a Meckel’s diverticulum have been reported [6–13, 16, 17]. The exact mechanism of perforation in patients with GIST within Meckel’s diverticula has not been fully described in the literature. The possible explanations include tumor necrosis, increased intraluminal pressure secondary to distal bowel obstruction, and tumor invasion into the muscularis propria, replacing the gut wall [17].

The standard surgical treatment of symptomatic Meckel’s diverticula is diverticulectomy, unless the indication for operative management is bleeding, in which case segmental resection of the ileum that includes both the diverticulum and the adjacent ileal ulcer should be performed [1]. In addition, however, segmental small bowel resection also should be considered in cases of perforation due to the chances of tumor within the diverticula.

## Conclusions

While gastrointestinal stromal tumor is a rare finding in Meckel’s diverticula, the potential for the coexistence of this and other tumors suggests that segmental resection of the small bowel should be considered in the treatment of perforated Meckel’s diverticulum.

## Consent

Written informed consent was obtained from the patient for publication of this Case Report and any accompanying images. A copy of the written consent is available for review by the Editor-in-Chief of this journal.

## Abbreviations

CD, cluster of differentiation; CT, computed tomography; GIST, gastrointestinal stromal tumor; SMA, smooth muscle actin
